# Association of Antidiabetic Medication Regimens and Medication Adherence With HbA1c Reduction in Type 2 Diabetic Patients: A Retrospective Study

**DOI:** 10.7759/cureus.92853

**Published:** 2025-09-21

**Authors:** Naveen Shaikh, Najma Qureshi, Maryam Junaid Qureshi, Zorez Rashid Mian, Haris Saeed, Muhammad Azzem Rana, Muhammad Ali

**Affiliations:** 1 Family Medicine, Aga Khan University, Karachi, PAK; 2 Internal Medicine, Sher-I-Kashmir Institute of Medical Sciences, Srinagar, IND; 3 Internal Medicine, Sir Ganga Ram Hospital, Lahore, PAK; 4 Internal Medicine, CMH (Combined Military Hospitals) Lahore Medical College and Institute of Dentistry, Lahore, PAK; 5 Internal Medicine, THQ (Tehsil Head Quarter) Hospital, Muridke, PAK; 6 Internal Medicine, Shalamar Medical and Dental College, Lahore, PAK

**Keywords:** antidiabetic medication, body mass index, fasting blood glucose levels, glycemic control, type 2 diabetes mellitus

## Abstract

Background

Effective management of type 2 diabetes mellitus (T2DM) requires not only appropriate pharmacological therapy but also consistent adherence to prescribed regimens. However, data on how different treatment regimens and adherence levels influence glycemic control in local populations are limited.

Objective

This study aimed to evaluate the impact of different medication regimens and adherence levels on glycemic control, as measured by glycated hemoglobin (HbA1c) and fasting blood glucose (FBG) in T2DM patients at a tertiary care hospital in Lahore.

Methods

This retrospective study was conducted at Combined Military Hospital, Lahore, Pakistan, from May 2023 to May 2025, including 355 patients with T2DM. Patients were categorized by regimen type: Group 1 (oral therapy), Group 2 (injectable therapy), and Group 3 (combination therapy). Medication adherence was classified into three groups based on compliance with prescription refill data and follow-up documentation: High (≥80%), Medium (50-79%), and Low (<50%). Clinical and demographic data were extracted from electronic health records. Outcomes included HbA1c, FBG, and body mass index (BMI) at six months. Statistical analyses included chi-square for categorical outcomes and analysis of variance (ANOVA) for continuous outcomes. A p-value <0.05 was considered significant.

Results

By regimen, Group 3 (combination therapy) demonstrated the greatest reductions in HbA1c (−2.0% ± 1.3%), FBG (−54.3 ± 40.2 mg/dL), and BMI (−1.4 ± 1.5 kg/m²), compared to Group 2 (−1.7% ± 1.1%, −42.7 ± 34.0 mg/dL, −1.1 ± 1.3 kg/m²) and Group 1 (−1.3% ± 0.9%, −35.2 ± 27.4 mg/dL, −0.6 ± 0.9 kg/m²) (p < 0.05). By adherence, high-adherence patients achieved lower mean HbA1c (7.0% vs. 7.8% vs. 8.6%), FBG (140 vs. 160 vs. 180 mg/dL), and BMI (29.2 vs. 30.5 vs. 32.0 kg/m²) compared with medium- and low-adherence groups. Adverse events were more common in the injectable and combination groups.

Conclusion

Both the choice of antidiabetic regimen and patient adherence significantly influence glycemic control. Combination therapy was associated with the greatest improvements in HbA1c, FBG, and BMI, particularly in patients with high adherence. Interventions targeting adherence improvement are essential to optimize therapeutic outcomes in T2DM.

## Introduction

Type 2 diabetes mellitus (T2DM) is a multifactorial, chronic metabolic disorder that represents a significant global health burden. Estimates provided by the International Diabetes Federation confirm that T2DM is noted in more than 460 million adult individuals around the globe, and this figure is also likely to be extremely large in the upcoming years [1]. The disease is marked by insulin resistance, where the cells of the body are responsive to higher insulin, and the beta-cell abnormality, where the insulin is poorly secreted. These pathophysiological alterations lead to hyperglycemia, which is a characteristic of diabetes that predisposes one to the development of chronic complications, which include retinopathy, nephropathy, cardiovascular disease, and neuropathy [2]. The key desired outcome of T2DM management is the achievement of optimal control of their blood glucose levels and the prevention of severe long-term complications. Glycated hemoglobin (HbA1c) is one of the most significant biomarkers to keep track of glycemic control [3]. HbA1c is an average measure of the ability of an individual to handle their blood glucose levels over a time period of 2-3 months and therefore serves as a good indicator of long-term glucose control. The American Diabetes Association (ADA) establishes a target HbA1c of less than 7% in the majority of adults with T2DM because the cutoff used to have a lower risk of microvascular and macrovascular complications [4]. Nevertheless, the realization of this aim necessitates the use of personalized plans; one size/type does not fit all regarding treating diabetes [5].

T2DM control usually consists of a rather balanced pattern of lifestyle measures and pharmacological management. Lifestyle intervention, which includes dietary alterations, active physical activity, and betterment of physical exercises, forms the foundation of the management of blood glucose [6]. Nevertheless, in the case that lifestyle changes are not enough, medication should be added. There is a great diversity in antidiabetic drugs, all with different modes of action that affect different dimensions of glucose metabolism. The most frequently used drugs are metformin, sulfonylureas, thiazolidinediones, dipeptidyl peptidase-4 (DPP-4) inhibitors, and, more recently, glucagon-like peptide-1 (GLP-1) receptor agonists and sodium-glucose cotransporter-2 (SGLT-2) inhibitors. Insulin, although not new, has long been an established and essential therapy for T2DM and remains the mainstay for patients who fail to achieve glycemic control with oral agents or present with advanced disease. According to the ADA Standards of Medical Care in Diabetes 2025, metformin remains the first-line pharmacological treatment for most patients with T2DM, unless contraindicated [7]. Metformin acts primarily by reducing hepatic glucose production and improving peripheral insulin sensitivity. It is associated with a significant reduction in HbA1c, but may be insufficient in patients with advanced disease or marked insulin resistance. Sulfonylureas are popularly prescribed as they stimulate insulin secretion in pancreatic beta cells, but they also carry a risk of hypoglycemia and are associated with weight gain, which may limit their long-term use [8].

Newer agents, including GLP-1 receptor agonists and SGLT-2 inhibitors, not only improve glycemic control but also provide well-documented cardiometabolic benefits. GLP-1 receptor agonists (e.g., liraglutide, semaglutide, dulaglutide) have demonstrated clinically significant weight loss, reductions in systolic blood pressure, and improved cardiovascular outcomes. The LEADER trial (liraglutide) and SUSTAIN-6 trial (semaglutide) showed significant reductions in major adverse cardiovascular events (MACE) in patients with T2DM and established cardiovascular disease [9,10]. SGLT-2 inhibitors (e.g., empagliflozin, dapagliflozin, canagliflozin) have proven benefits beyond glucose lowering. The EMPA-REG OUTCOME trial (empagliflozin) showed a 38% relative risk reduction in cardiovascular death, while the CANVAS program (canagliflozin) demonstrated reductions in MACE [11,12]. Additionally, the DAPA-HF trial confirmed that dapagliflozin reduced the risk of worsening heart failure or cardiovascular death, and the CREDENCE trial established its role in slowing the progression of chronic kidney disease (CKD) [13,14]. According to the ADA 2025 Standards of Medical Care, both GLP-1 receptor agonists and SGLT-2 inhibitors are recommended in patients with T2DM and established atherosclerotic cardiovascular disease (ASCVD), heart failure, or CKD, independent of baseline HbA1c or metformin use [15]. Recent developments have shown that these newer drugs have led to more individualized methods of treatment in dealing with patients [16]. The use of GLP-1 receptor agonists, liraglutide and semaglutide, raises insulin levels in response to food, suppresses glucagon release, and slows down the digestion of food, which leads to a healthy reduction in blood glucose levels and weight. SGLT-2 inhibitors, which include empagliflozin and canagliflozin, block the reabsorption of glucose produced by the kidneys and produce urinary loss of glucose [17]. Such agents have also been implicated in a decrease in the occurrence of cardiovascular problems and progression of diabetic kidney disease, which is especially favorable to patients who often exhibit more than one health problem [18]. Although it is possible today to find numerous pharmacological methods of regulating glycemic control, this goal is difficult to reach in many cases. Treatment is always complicated by factors like patient compliance, comorbid factors, and the existence of complications of diabetes [19].

Objective

This study aimed to evaluate the impact of different medication regimens and adherence levels on glycemic control, as measured by HbA1c and fasting blood glucose (FBG) in T2DM patients at a tertiary care hospital in Lahore.

## Materials and methods

Methodology

This retrospective study was conducted at Combined Military Hospital, Lahore, Pakistan, from May 2023 to May 2025. A total of 355 patients diagnosed with T2DM were included in the study. The sample size was determined based on the availability of medical records that met the inclusion criteria.

Inclusion and exclusion criteria

Participants were eligible for inclusion in the study if they were adults aged between 18 and 75 years and had been diagnosed with T2DM for at least six months prior to the study. Participants were required to have a baseline HbA1c level of ≥7% either at the time of enrollment or during any follow-up visit. Additionally, they must have been on stable antidiabetic medication regimens for a minimum of three months before the initiation of the study.

The exclusion criteria included patients diagnosed with type 1 diabetes, gestational diabetes, or other specific forms of diabetes, such as maturity-onset diabetes of the young (MODY), pancreatogenic diabetes, or steroid-induced diabetes. Individuals with CKD stage IV or higher (estimated glomerular filtration rate [eGFR] <30 mL/min/1.73 m²), decompensated liver disease (Child-Pugh class C), or advanced cardiovascular disease (New York Heart Association [NYHA] class III-IV heart failure or recent myocardial infarction within six months) were excluded. Furthermore, pregnant or breastfeeding women and patients with incomplete or missing data regarding HbA1c levels or medication regimens were not considered for inclusion in the study.

Data collection

Data were retrospectively extracted from the patients’ electronic health records (EHRs), focusing on a range of clinical and demographic variables. Two independent reviewers cross-verified the extracted data to minimize transcription errors, and discrepancies were resolved through consensus with a third reviewer. Demographic information included age, gender, and comorbid conditions such as hypertension and dyslipidemia. Clinical data included the duration of diabetes, baseline HbA1c levels, body mass index (BMI), and FBG levels. Baseline HbA1c was defined as the most recent value recorded within three months prior to initiation of the documented antidiabetic regimen, while follow-up HbA1c was assessed at six months (±1 month) after therapy initiation. HbA1c levels were measured using standardized high-performance liquid chromatography (HPLC), with internal laboratory quality controls in place to ensure accuracy and comparability across all samples.

The type of medication regimen prescribed, the duration of therapy, and any changes in the treatment regimen during the study period were also recorded. In cases of missing HbA1c or FBG values, a complete case analysis was applied; patients with incomplete key outcome data were excluded from the final analysis. The primary outcome measure of the study was the change in HbA1c levels from baseline to six months after regimen initiation. Secondary outcomes included changes in FBG levels, BMI, and the occurrence of any adverse events related to the medications. Hypoglycemia was defined as a documented plasma glucose <70 mg/dL (3.9 mmol/L) and severe hypoglycemia as an event requiring external assistance. Gastrointestinal side effects were defined as persistent nausea, vomiting, or diarrhea requiring treatment modification.

Statistical analysis

The collected data were analyzed using statistical software SPSS version 26.0 (IBM Corp., Armonk, NY). Descriptive statistics were used to summarize the demographic and clinical characteristics of the study cohort. The primary outcome, HbA1c reduction, was analyzed using paired t-tests or repeated-measures ANOVA to compare changes in HbA1c levels between the three medication groups over time. Post-hoc pairwise comparisons were conducted to assess significant differences between the groups. Multivariate regression analysis was also performed to adjust for potential confounding factors such as age, duration of diabetes, and the presence of comorbid conditions. A p-value of ≤0.05 was considered statistically significant.

## Results

The study population consisted of 355 patients with T2DM, with a mean age of 58.4 ± 9.6 years, ranging from 38 to 74 years. Of the total participants, 54.9% were male and 45.1% were female. The mean duration of diabetes was 9.3 ± 5.2 years. The baseline HbA1c level was 8.2% ± 1.4%, with 35.5% of patients having hypertension, 21.2% having dyslipidemia, and 12.8% having CKD. The mean BMI was 30.2 ± 4.5 kg/m², and the mean FBG was 156.4 ± 35.8 mg/dL (Table 1).

**Table 1:** Demographic, Baseline Clinical Characteristics, and Medication Distribution of the Study Population Data are represented as mean ± SD or percentages. HbA1c, glycated hemoglobin; CKD, chronic kidney disease; BMI, body mass index; FBG, fasting blood glucose.

Characteristic	Value (n = 355)
Mean age (years)	58.4 ± 9.6
Age range (years)	38-74
Gender	
- Male	54.9%
- Female	45.1%
Mean duration of diabetes (years)	9.3 ± 5.2
Baseline HbA1c (%)	8.2 ± 1.4
Comorbidities	
- Hypertension	35.5%
- Dyslipidemia	21.2%
- CKD	12.8%
Mean BMI	30.2 ± 4.5
Mean FBG (mg/dL)	156.4 ± 35.8
Mean systolic blood pressure (mmHg)	132.1 ± 14.6
Mean diastolic blood pressure (mmHg)	84.3 ± 9.7
Medication regimens	
- Group 1: Oral antidiabetic medications	175 patients (49.3%)
- Group 2: Injectable medications	125 patients (35.2%)
- Group 3: Combination therapy	55 patients (15.5%)

In Group 1 (Oral Antidiabetic Medications), metformin was the most commonly used medication, prescribed to 64% of patients, followed by sulfonylureas in 36% of patients. Group 2 (Injectable Medications) had 32.8% of patients prescribed GLP-1 receptor agonists, with 67.2% on insulin. Group 3 (Combination Therapy) included patients prescribed a combination of medications: 32.7% of patients received metformin, 29.1% received sulfonylureas, and 41.8% received insulin, with some patients using combinations such as metformin and GLP-1 receptor agonists (Table 2).

**Table 2:** Distribution of Antidiabetic Medications at Baseline Values represent the number of patients, with the percentage values inside parentheses. GLP-1, glucagon-like peptide-1.

Medication Type	Group 1: Oral Antidiabetic Medications (n = 175)	Group 2: Injectable Medications (n = 125)	Group 3: Combination Therapy (n = 55)
Metformin	112 (64.0%)	0 (0%)	18 (32.7%)
Sulfonylureas	63 (36.0%)	0 (0%)	16 (29.1%)
GLP-1 receptor agonists	0 (0%)	41 (32.8%)	14 (25.5%)
Insulin	0 (0%)	84 (67.2%)	23 (41.8%)
Combination of metformin and insulin	0 (0%)	0 (0%)	7 (12.7%)
Combination of metformin and GLP-1 agonist	0 (0%)	0 (0%)	13 (23.6%)
Other combinations	0 (0%)	0 (0%)	5 (9.1%)

At the six-month follow-up (defined as 6 months ± 1 month to account for variability in visit scheduling), the mean HbA1c was 7.0% ± 1.3% in Group 1, 6.8% ± 1.4% in Group 2, and 6.2% ± 1.2% in Group 3. A higher percentage of patients in Group 3 (76.4%) achieved an HbA1c <7%, compared to 60.0% in Group 1 and 65.6% in Group 2. Similarly, the percentage of patients achieving an HbA1c <6.5% was highest in Group 3 (61.8%), followed by Group 2 (52.8%) and Group 1 (42.3%). The mean FBG reduction at the last recorded visit within the follow-up period was 145.3 ± 33.6 mg/dL in Group 1, 140.2 ± 32.1 mg/dL in Group 2, and 132.5 ± 30.8 mg/dL in Group 3. Patients were typically evaluated at baseline, three months, and six months, although the last available measurement within this window was used for analysis (Table 3).

**Table 3:** Follow-Up Data on HbA1c and FBG at Last Visit Data are shown as mean ± SD or percentages. HbA1c, glycated hemoglobin; FBG, fasting blood glucose.

Measurement	Group 1: Oral Antidiabetic Medications (n = 175)	Group 2: Injectable Medications (n = 125)	Group 3: Combination Therapy (n = 55)
Mean HbA1c at last visit (%)	7.0 ± 1.3	6.8 ± 1.4	6.2 ± 1.2
Mean FBG at last visit (mg/dL)	145.3 ± 33.6	140.2 ± 32.1	132.5 ± 30.8
Percent of patients achieving HbA1c <7%	60.0	65.6	76.4
Percent of patients achieving HbA1c <6.5%	42.3	52.8	61.8

Gastrointestinal disturbances were most frequent in the injectable group (28.0%), with a significantly higher risk compared to oral therapy (risk ratio [RR]: 2.72, 95% CI: 1.62-4.58, p < 0.001). Hypoglycemia was most prevalent in the combination therapy group (11.5%), which showed a nearly fourfold increased risk compared to oral agents (RR: 3.82, 95% CI: 1.21-12.03, p = 0.025). Urinary tract infections (UTIs) and genital infections occurred rarely but were observed more often in injectable and combination groups, with statistical significance reached for UTIs (p = 0.029 and p = 0.013, respectively). Weight gain was most common in the combination group (6.3%), but the increase did not reach statistical significance compared to oral therapy (p = 0.090) (Table 4).

**Table 4:** Comparative Risk of Adverse Events by Medication Regimen Data are shown as n (%). Group 1 (oral antidiabetic medications) was used as the reference category. “—” indicates risk ratio not calculable due to zero events in the reference group. Statistical significance is defined as p < 0.05. RR, risk ratio; CI, confidence interval; GI, gastrointestinal; UTIs, urinary tract infections.

Adverse Event	Group 1: Oral (n = 175)	Group 2: Injectable (n = 125)	Group 3: Combination (n = 55)	RR (2 vs 1) (95% CI)	p-Value	RR (3 vs 1) (95% CI)	p-Value
GI disturbances	18 (10.5%)	35 (28.0%)	5 (8.6%)	2.72 (1.62-4.58)	<0.001	0.88 (0.34-2.27)	1.000
Hypoglycemia	5 (2.6%)	6 (4.9%)	6 (11.5%)	1.68 (0.52-5.38)	0.535	3.82 (1.21-12.03)	0.025
UTIs	0 (0.0%)	4 (3.1%)	3 (5.7%)	—	0.029	—	0.013
Genital infections	0 (0.0%)	3 (2.4%)	2 (4.2%)	—	0.071	—	0.056
Weight gain	2 (1.2%)	3 (2.4%)	3 (6.3%)	2.10 (0.36-12.38)	0.653	4.77 (0.82-27.84)	0.090

The results of changes in clinical outcomes indicated that Group 3 (Combination Therapy) had the largest reductions in HbA1c (2.0% ± 1.3%), FBG (54.3 ± 40.2 mg/dL), and BMI (1.4 ± 1.5 kg/m²), followed by Group 2 (Injectable Medications) with reductions of 1.7% ± 1.1% in HbA1c, 42.7 ± 34.0 mg/dL in FBG, and 1.1 ± 1.3 kg/m² in BMI. Group 1 (Oral Antidiabetic Medications) showed the least reductions in all three outcomes: 1.3% ± 0.9% in HbA1c, 35.2 ± 27.4 mg/dL in FBG, and 0.6 ± 0.9 kg/m² in BMI (Table 5).

**Table 5:** Changes in Clinical Outcomes (HbA1c, FBG, BMI) by Medication Regimen Data are shown as mean ± SD and adjusted mean change with 95% CI. Significance level: p < 0.05. HbA1c, glycated hemoglobin; FBG, fasting blood glucose; BMI, body mass index; CI, confidence interval.

Medication Regimen	HbA1c Reduction (Mean ± SD)	Adjusted Mean Change (95% CI)	p-Value	FBG Reduction (mg/dL, Mean ± SD)	Adjusted Mean Change (95% CI)	p-Value	BMI Reduction (kg/m², Mean ± SD)	Adjusted Mean Change (95% CI)	p-Value
Group 1: Oral (n = 175)	1.3 ± 0.9	1.25 (1.08-1.42)	—	35.2 ± 27.4	34.7 (30.5-38.9)	—	0.6 ± 0.9	0.58 (0.45-0.71)	—
Group 2: Injectable (n = 125)	1.7 ± 1.1	1.68 (1.49-1.87)	0.02 vs G1	42.7 ± 34.0	42.1 (37.2-47.0)	0.03 vs G1	1.1 ± 1.3	1.09 (0.92-1.26)	0.01 vs G1
Group 3: Combination (n = 55)	2.0 ± 1.3	1.98 (1.75-2.21)	0.03 vs G1, 0.04 vs G2	54.3 ± 40.2	53.7 (47.2-60.2)	0.04 vs G1, 0.05 vs G2	1.4 ± 1.5	1.38 (1.12-1.64)	0.02 vs G2

Patients with high adherence to their prescribed antidiabetic regimen achieved significantly better glycemic control, as evidenced by lower final HbA1c and FBG, greater HbA1c reduction, and lower BMI compared to medium and low adherence groups (all p < 0.01) (Table 6).

**Table 6:** Clinical Outcomes by Medication Adherence *Statistically significant at p < 0.05. Adherence level was based on compliance & follow-up notes: High: Took ≥80% of prescribed doses; regular follow-up visits; documented good compliance. Medium: Took 50-79% of prescribed doses; occasional missed visits; partial compliance. Low: Took <50% of prescribed doses; frequent missed visits; poor compliance. HbA1c, glycated hemoglobin; FBG, fasting blood glucose; BMI, body mass index.

Outcome	High Adherence (n = 145)	Medium Adherence (n = 120)	Low Adherence (n = 90)	p-Value
Final HbA1c (%)	7.0 ± 1.1	7.8 ± 1.3	8.6 ± 1.5	<0.001*
HbA1c reduction (%)	−1.9 ± 1.2	−1.3 ± 1.0	−0.8 ± 0.9	<0.001*
Final FBG (mg/dL)	140 ± 28	160 ± 33	180 ± 35	<0.001*
Final BMI (kg/m²)	29.2 ± 4.1	30.5 ± 4.3	32.0 ± 4.6	0.002*

## Discussion

In this retrospective study of 355 adults with T2DM, we evaluated the effectiveness of oral medications, injectable therapies, and combination regimens on glycemic control, FBG, and BMI. We also assessed the impact of medication adherence on clinical outcomes and examined adverse event profiles across treatment groups.

Our findings demonstrated clear differences in HbA1c among adherence groups. The mean HbA1c was 7.0%, 7.8%, and 8.6% in the high-, medium-, and low-adherence groups, respectively. These results align with prior literature, indicating that poor adherence significantly contributes to suboptimal glycemic control [20]. Elevated HbA1c in non-adherent patients has been associated with increased risks of microvascular complications, including retinopathy and nephropathy [21,22]. Our study supports this relationship, as high adherence correlated with more favorable glycemic control and potentially lower risk of complications.

FBG levels also exhibited a strong relationship with adherence. The mean FBG was 140 mg/dL in the high-adherence group, compared to 160 mg/dL and 180 mg/dL in the medium- and low-adherence groups, respectively. These differences were statistically significant and underscore the critical role of regular antidiabetic medication intake in maintaining optimal glucose levels [23,24].

BMI, an important marker of metabolic health, differed significantly among adherence groups. Multivariate analysis revealed a mean BMI of 29.2 kg/m² in the high-adherence group versus 32.0 kg/m² in the low-adherence group [25]. Non-adherence may coincide with unhealthy lifestyle behaviors, such as poor diet and sedentary activity, contributing to weight gain [26]. Conversely, patients with higher adherence may have benefited from GLP-1 receptor agonists or SGLT-2 inhibitors, which are known to promote weight reduction.

Regarding treatment regimens, patients receiving combination therapy (oral plus injectable) achieved the largest reductions in HbA1c (1.98%), FBG (53.7 mg/dL), and BMI (1.38 kg/m²) over a 3-6-month follow-up period. These reductions were statistically significant, as confirmed by analysis of covariance (ANCOVA) with post-hoc contrasts, and are clinically meaningful, as even a ~2% decrease in HbA1c reduces the risk of microvascular complications and cardiovascular events. Injectable therapies alone provided greater improvements than oral medications alone, highlighting the efficacy of GLP-1 receptor agonists and insulin for patients inadequately controlled on oral therapy.

The incidence of adverse events varied by regimen. Gastrointestinal disturbances were most common with injectable therapies (28.0%), consistent with known effects of GLP-1 receptor agonists. Hypoglycemia occurred more frequently in combination therapy (11.5%), reflecting the additive effects of insulin and oral agents. UTIs and genital infections were predominantly observed in Groups 2 and 3, likely related to SGLT-2 inhibitor use. Relative risk analysis showed a significantly higher risk of hypoglycemia in combination therapy compared to oral therapy alone (RR: 3.82, 95% CI: 1.21-12.03, p = 0.025). While most adverse events were mild to moderate, these findings highlight the importance of careful patient selection, monitoring, and dose adjustment.

Overall, the differences in HbA1c, FBG, and BMI among adherence groups emphasize the critical importance of medication adherence in T2DM management. Healthcare providers should implement strategies to improve adherence, such as patient education, reducing barriers to access, simplifying regimens, and conducting regular follow-up [27,28]. High adherence enhances treatment effectiveness across all regimens, and combination therapy may provide superior glycemic control for patients inadequately managed on oral or injectable therapy alone. However, clinicians must balance efficacy with safety, particularly considering the increased hypoglycemia risk associated with combination therapy.

This study has limitations. As a retrospective analysis, it relied on existing records, which may be incomplete or inaccurate and varied in follow-up intervals (3-6 months), limiting causal inference. Medication adherence was categorized into High, Medium, and Low groups, but the assessment method was not standardized, introducing potential recall bias. Rare adverse events were difficult to evaluate due to small subgroup sizes, and unmeasured confounders may have influenced results. Despite these limitations, this study provides valuable real-world insights into how different antidiabetic regimens and adherence levels affect glycemic control, BMI, and safety outcomes in patients with T2DM.

## Conclusions

It is concluded that medication adherence is strongly associated with improved clinical outcomes in patients with T2DM. In this study, patients in the Combination Therapy group (Group 3), who demonstrated higher adherence, experienced the largest reductions in HbA1c, FBG, and BMI compared to those in the Oral Antidiabetic Medications (Group 1) and Injectable Medications (Group 2) groups. These findings suggest that combining oral and injectable therapies may be linked to enhanced glycemic control and weight management, potentially reflecting the complementary effects of different drug classes.
